# Genome-wide profiling of host-encoded circular RNAs highlights their potential role during the Japanese encephalitis virus-induced neuroinflammatory response

**DOI:** 10.1186/s12864-020-06822-5

**Published:** 2020-06-18

**Authors:** Yunchuan Li, Usama Ashraf, Zheng Chen, Dengyuan Zhou, Muhammad Imran, Jing Ye, Huanchun Chen, Shengbo Cao

**Affiliations:** 1 State Key Laboratory of Agricultural Microbiology, Huazhong Agricultural University, Wuhan, 430070 Hubei China; 2 Laboratory of Animal Virology, College of Veterinary Medicine, Huazhong Agricultural University, Wuhan, 430070 Hubei China; 3 The Cooperative Innovation Center for Sustainable Pig Production, Huazhong Agricultural University, Wuhan, 430070 Hubei China

**Keywords:** Japanese encephalitis virus, Neuroinflammation, circRNA, ceRNA

## Abstract

**Background:**

Japanese encephalitis virus (JEV) is one of the common causes of acute encephalitis in humans. Japanese encephalitis is characterized by the uncontrolled release of inflammatory cytokines, which ultimately results in neuronal cell damage. In recent years, with the advancement of high-throughput sequencing technology, studies have shown that circRNAs, by competing with endogenous miRNAs, play a vital role in the pathology of CNS diseases. However, it is unknown whether circRNAs participate in JEV-induced neuroinflammation.

**Results:**

By employing Illumina RNA-sequencing, we identified 180 circRNAs and 58 miRNAs that showed significant differential expression in JEV-infected mice brain tissues. The functional enrichment analyses revealed that these differentially regulated circRNAs were predominantly related to neurotransmission, histone modifications, transcription misregulation, and inflammation-associated calcium signaling pathway. Our established competing endogenous RNA (ceRNA) interaction network suggested the correlation of several circRNAs, miRNAs, and mRNAs in regulating the inflammatory response during JEV infection. Among the predicted interactions, the correlation between circ_0000220, miR-326-3p, and BCL3/MK2/TRIM25 mRNAs was experimentally validated by knockdown or overexpression of the non-coding RNA entities in cultured mouse microglia. The knockdown of circ_0000220 or overexpression of miR-326-3p caused a lower production of JEV-induced inflammatory cytokines.

**Conclusions:**

Conclusively, our study provides new insights into the host response to JEV infection and proposes the circRNA-targeting therapeutic interventions to rein in Japanese encephalitis.

## Background

Japanese encephalitis virus (JEV) is a mosquito-borne flavivirus that belongs to the *Flaviviridae* family, and is closely related to West Nile virus, Zika virus, and dengue virus [1]. JEV is the causative agent of Japanese encephalitis (JE), which is mainly prevalent in several regions of eastern Asia, southeastern Asia, and Oceania [2]. According to an estimation, 35,000–50,000 cases of JE are reported annually in these regions with 10,000 death, and ~ 50% of the recovered patients undergo permanent neurological sequelae [3]. JEV neuropathogenesis is characterized by the activation of a robust inflammatory response and subsequent neuronal cell death. The key factor in JEV-induced neuroinflammation is the unbridled activation of microglia, which release high levels of inflammatory cytokines and chemokines that include tumor necrosis factor-α (TNF-α), interleukin (IL)-1β, IL-6, and chemokine (C-C motif) ligand 5 (CCL5) [4, 5]. Several pieces of evidence showed that microglia can be directly infected with JEV and serve as a virus reservoir [6]; therefore, targeting of microglia by JEV is a pivotal step to induce neuroinflammation. Albeit several molecular mechanisms of JEV pathogenesis have been unveiled in the last few years, further studies are needed to advance the understanding of JEV pathogenesis.

Circular RNAs (circRNAs) are identified as a new class of endogenous non-coding RNAs, produced as a result of the back-splicing process [7]. They have been found extensively dispersed among organisms and show tissue/cell-type specificity [8]. Many studies have demonstrated that several biological processes, including gene regulation, RNA metabolism, protein assembly and trafficking, and cell division, are tightly regulated by circRNAs [9–11]. Anomalous regulation of circRNAs has also been associated with a variety of pathologies, especially cancers and autoimmunity [12, 13]. Moreover, recent RNA-seq studies showed that the expression of a large number of circRNAs undergoes alteration during the replication of the herpes virus, human immunodeficiency virus, and avian leukosis virus [13–15]. Notably, circRNAs carry conserved sequence motifs that are a complement to the sequences of microRNAs (miRNAs), thus, they can alter the activity of miRNAs by acting as miRNA-sponges [16]. Albeit miRNAs are known to participate in the JEV pathogenesis, the role of host-encoded circRNAs in mediating the JEV-induced neuroinflammation is undetermined [17, 18].

In this study, the genome-wide transcriptomic profiling of circRNAs and miRNAs from JEV-infected mice brain tissues was performed using the Illumina sequencing. A large number of circRNAs and miRNAs were found to be differentially expressed upon JEV infection with their potential role in regulating the JEV-induced neuroinflammation. This study may provide new insights into the pathogenic mechanisms by which JEV causes neuropathogenesis.

## Results

### JEV infection alters the expression profile of circRNAs in mice brain

**Fig. 1:**
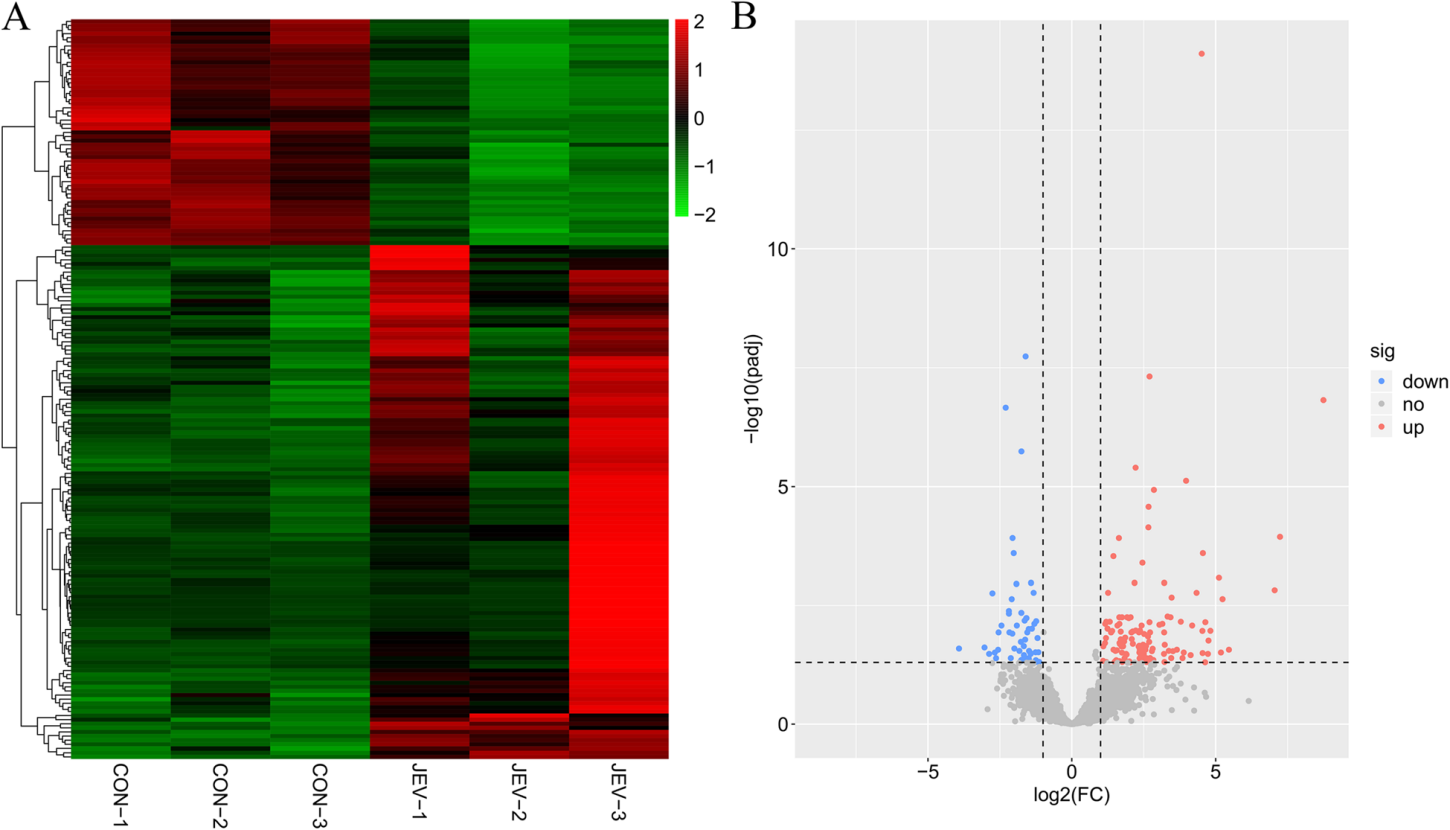
The expression profile of circRNAs in mice brain during JEV infection. **a** The hierarchical clustering of differently expressed circRNAs. The red color indicates up-regulation and the green color indicates down-regulation. **b** The volcano plot representing the circRNAs expression. The grey dots denote non-significantly expressed circRNAs. The up-regulated circRNAs are presented as red dots and the down-regulated circRNAs are presented as blue dots

Initially, we successfully established the mice model of JEV infection, which has been described previously by our laboratory [19]. To investigate the expression profile of circRNAs in mice brain infected with JEV, the rRNA- and linear RNA-depleted total RNAs extracted from JEV-infected or mock-infected brain tissue samples were employed for genome-wide sequencing. In total, we acquired ~ 85–150 million raw sequencing reads in infected or mock-infected samples. Subsequently, the clean reads were mapped to the mouse mm10 genome using the BWA-MEM alignment tool. To this end, 49,632 candidate circRNAs with more than one back-spliced read were obtained (Additional file 1: Table S1) through the CIRI v2.0.6 software [20]. In total, 180 significantly differentially regulated (fold change > = 2 and FDR < = 0.05) circRNAs were identified in JEV-infected mice brain samples compared to mock-infected samples. Among them, 125 circRNAs were found to be up-regulated, whereas 55 circRNAs showed a down-regulated expression pattern (Additional file 2: Table S2). The hierarchical clustering (Fig. 1a) and the volcano plot (Fig. 1b) showed the global regulatory pattern of circRNAs upon JEV infection.

### Properties of the circRNAs detected in JEV-infected and mock-infected mice brain

**Fig. 2:**
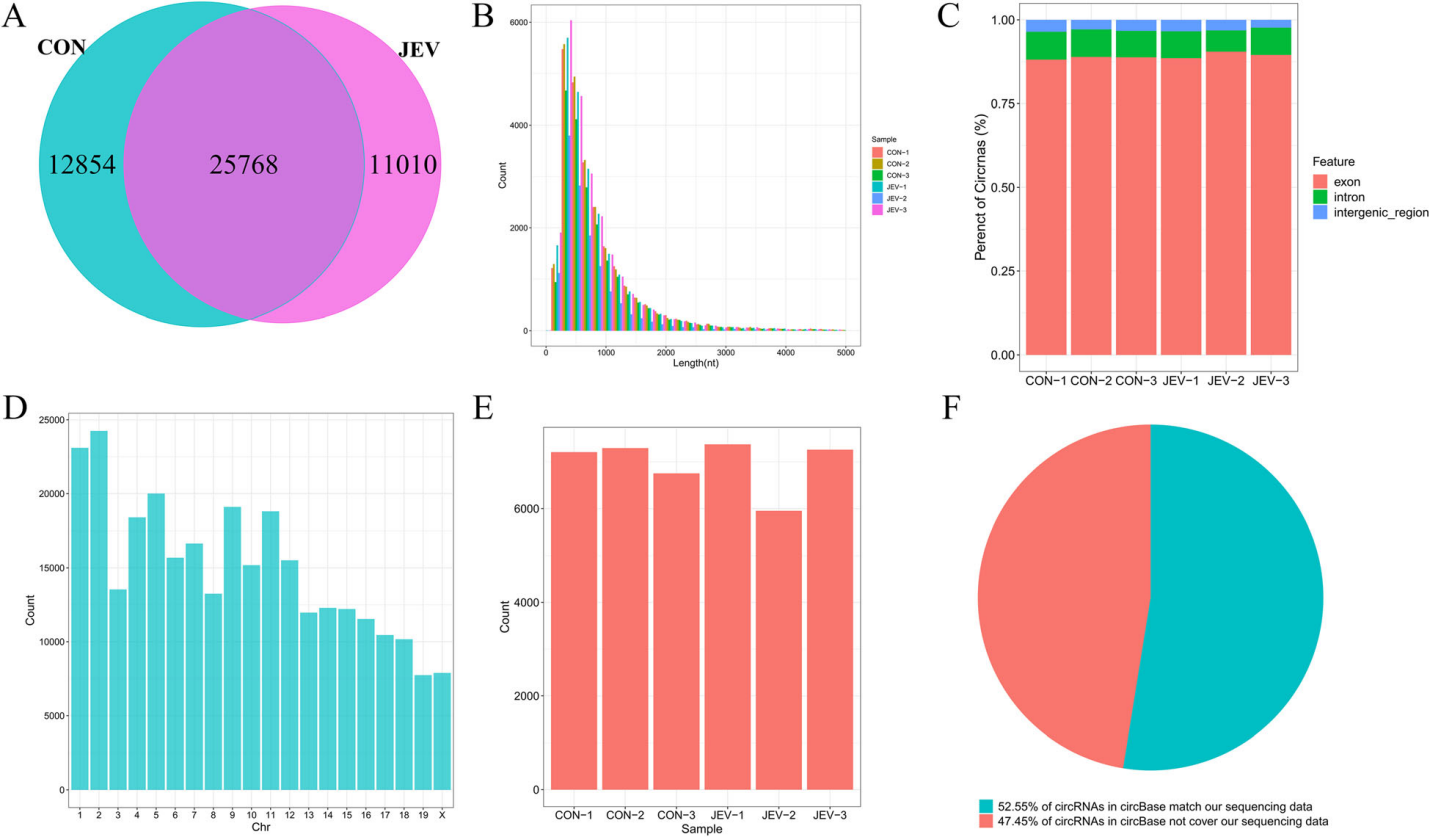
The genomic features of circRNAs detected in mice brain during JEV infection. **a** The comparison of all identified circRNAs between two experimental groups. **b** The length distribution statistics of circRNAs. **c** The genomic origin distribution of circRNAs. **d** The distribution of circRNAs on mouse chromosomes. **e** The number of detected circRNAs matched with circRNAs in the circBase database. **f** The proportion of detected circRNAs matched with circRNAs in the circBase database

Among the 49,632 detected circRNAs, 25,039 were observed to be common between both experimental groups, whereas 11,143 and 12,893 circRNAs were uniquely expressed in the JEV-infected and the mock-infected group, respectively (Fig. 2a). The distribution of circRNAs length was analyzed and displayed in Fig. 2b. Most of the circRNAs were observed shorter than 2000 nt, and the number of circRNAs was decreased with the increase in their sequence length. As it is reported that most of the circRNAs are generated from the exons [7], we next analyzed the genomic origin of the circRNAs identified in two experimental groups. In agreement with the previous studies, > 90% of circRNAs were originated from exons, whereas only a minute fraction of them derived from introns or intergenic regions (Fig. 2c). The identified circRNAs were widely distributed from chromosome 1 to chromosome X, and chromosomes 1 and 2 produced ~ 4000 circRNAs (Fig. 2d).

The circBase database incorporates circRNAs from several eukaryotic organisms including the *M. musculus* [21]. We compared 49,632 circRNAs, identified in our study, with the circBase circRNAs. Interestingly, ~ 6000 circRNAs detected in each sample were annotated in the circBase (Fig. 2e), and the remaining ones (~ 50%) appeared to be non-annotated which could be either novel or tissue/cell-specific (Fig. 2f).

### GO term and pathway enrichment analyses of differently expressed circRNAs’ parent genes

To determine the biological importance of differently expressed circRNAs, we performed GO term and pathway enrichment analyses by considering their parent genes. The biological process and molecular function classification revealed that these circRNAs were predominantly associated with neurotransmission, epigenetic modification, and ion channel activities (Additional file 3: Figure S1A and Additional file 4: Table S3). The cellular distribution analysis predicted their localization primarily in the synapses (Additional file 3: Figure S1A and Additional file 4: Table S3). Moreover, several crucial pathways, involving transcriptional regulation, phospholipase D/Phosphatidylinositol signaling, and inflammation-related calcium signaling, were found to be markedly triggered by differentially expressed circRNAs (Additional file 3: Figure S1B and Additional file 4: Table S3).

### Construction of the circRNA-miRNA-mRNA competing endogenous RNA (ceRNA) network

**Fig. 3:**
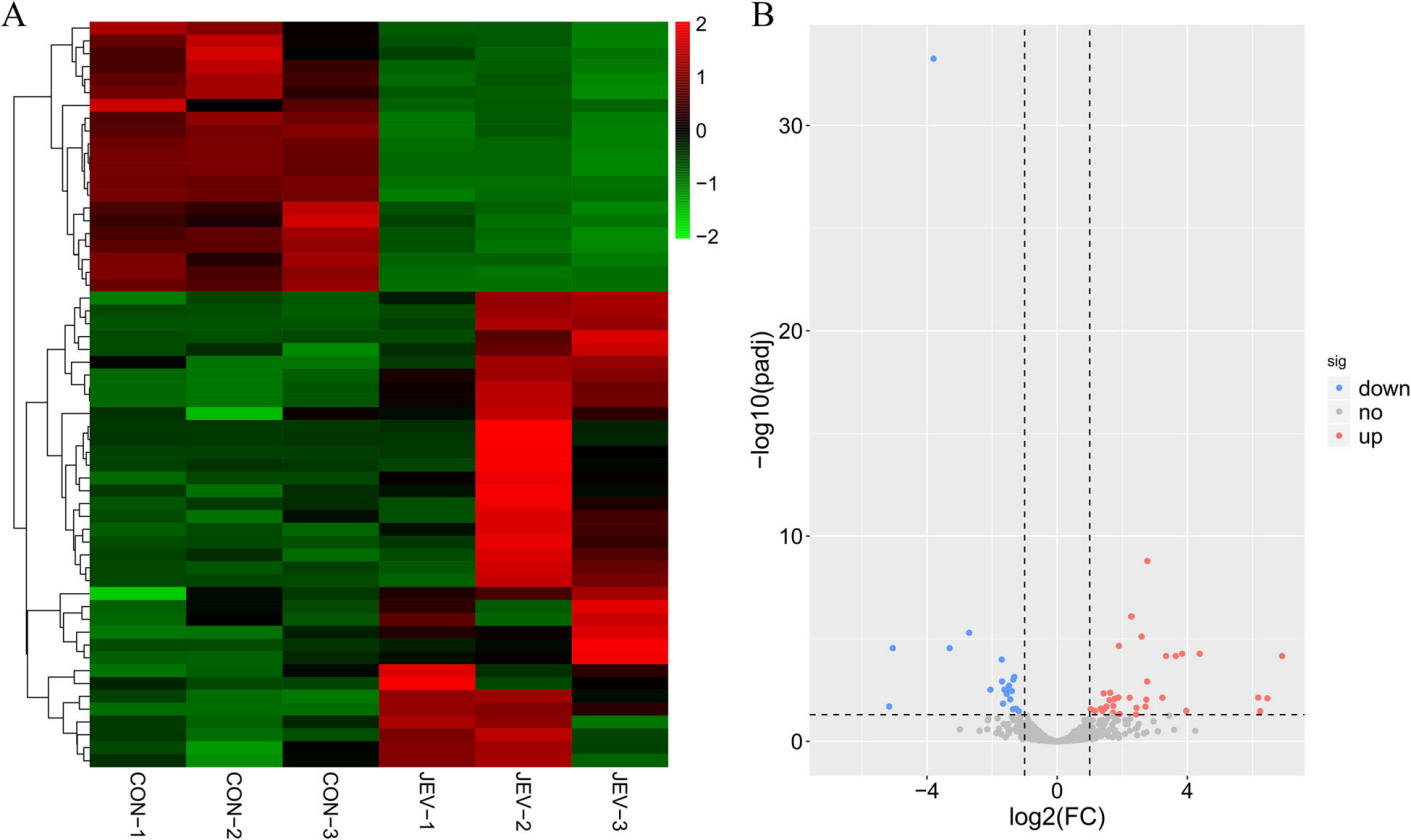
The expression profile of miRNAs in mice brain during JEV infection. **a** The hierarchical clustering of differently expressed miRNAs. The red color indicates up-regulation and the green color indicates down-regulation. **b** The volcano plot representing the miRNAs expression. The grey dots denote non-significantly expressed miRNAs. The up-regulated miRNAs are presented as red dots and the down-regulated miRNAs are presented as blue dots

Previous studies have shown that circRNAs can serve as miRNA sponges to regulate the expression of mRNAs [22, 23]. Our small RNA sequencing analysis of JEV-infected or mock-infected mice brain samples revealed a total of 1287 miRNAs detected in both experimental groups. Of these, 58 miRNAs showed a significant differential expression pattern (37 up-regulated and 21 down-regulated; fold change > = 2 and FDR < = 0.05) upon JEV infection when compared to mock-infected mice samples (Additional file 5: Table S4), as assessed through the R package limma [24]. The overall expression pattern of miRNAs induced by JEV infection is displayed by the hierarchical clustering (Fig. 3a) and the volcano plot (Fig. 3b).

**Fig. 4:**
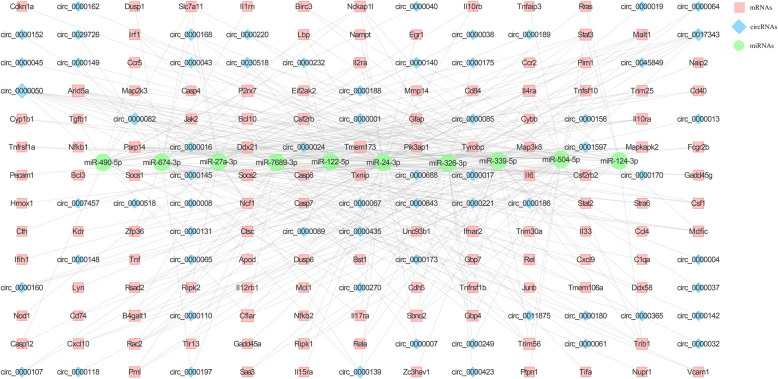
The inflammation-associated ceRNA network. The circles represent miRNAs, squares represent mRNAs, and rhombuses represent circRNAs

In combination with our previous data of microarray analysis of JEV-induced mRNAs expression in mice brain tissues [19] together with circRNAs and miRNAs expression data obtained in this study, we predicted the correlation of circRNAs, miRNAs, and mRNAs using the miRanda algorithm. In total, we detected 569 negatively regulated miRNA-circRNA pairs and 2719 negatively regulated miRNA-mRNA pairs (Additional file 6: Table S5). To determine the role of circRNAs in JEV-induced neuroinflammatory response, we chose those differentially expressed circRNAs that showed the potential to be involved in the inflammatory response, and subsequently, constructed the inflammation-related circRNA-miRNA-mRNA ceRNA network (Fig. 4). The list of the degree of interaction of circRNA-miRNA-mRNA in the ceRNA network is presented in Table 1.

**Table 1:** The top 10 degrees of ncRNAs in the inflammation-associated ceRNA network

miRNA	Degree	mRNA	Degree	circRNA	Degree
miR-24-3p	64	Cflar	10	circ_0000050	19
miR-326-3p	58	Arid5a	9	circ_0000082	12
miR-339-5p	52	Il4ra	7	circ_0017343	10
miR-7689-3p	40	P2rx7	7	circ_0000168	8
miR-504-5p	39	Sbno2	7	circ_0000139	8
miR-27a-3p	35	Socs2	7	circ_0000140	6
miR-674-3p	34	Trim56	7	circ_0030518	5
miR-122-5p	32	B4galt1	6	circ_0000017	5
miR-490-5p	24	Stat3	6	circ_0000001	5
miR-5129-3p	22	Trib1	6	circ_0001597	4

### Validation of the differentially expressed circRNAs and miRNAs

**Fig. 5:**
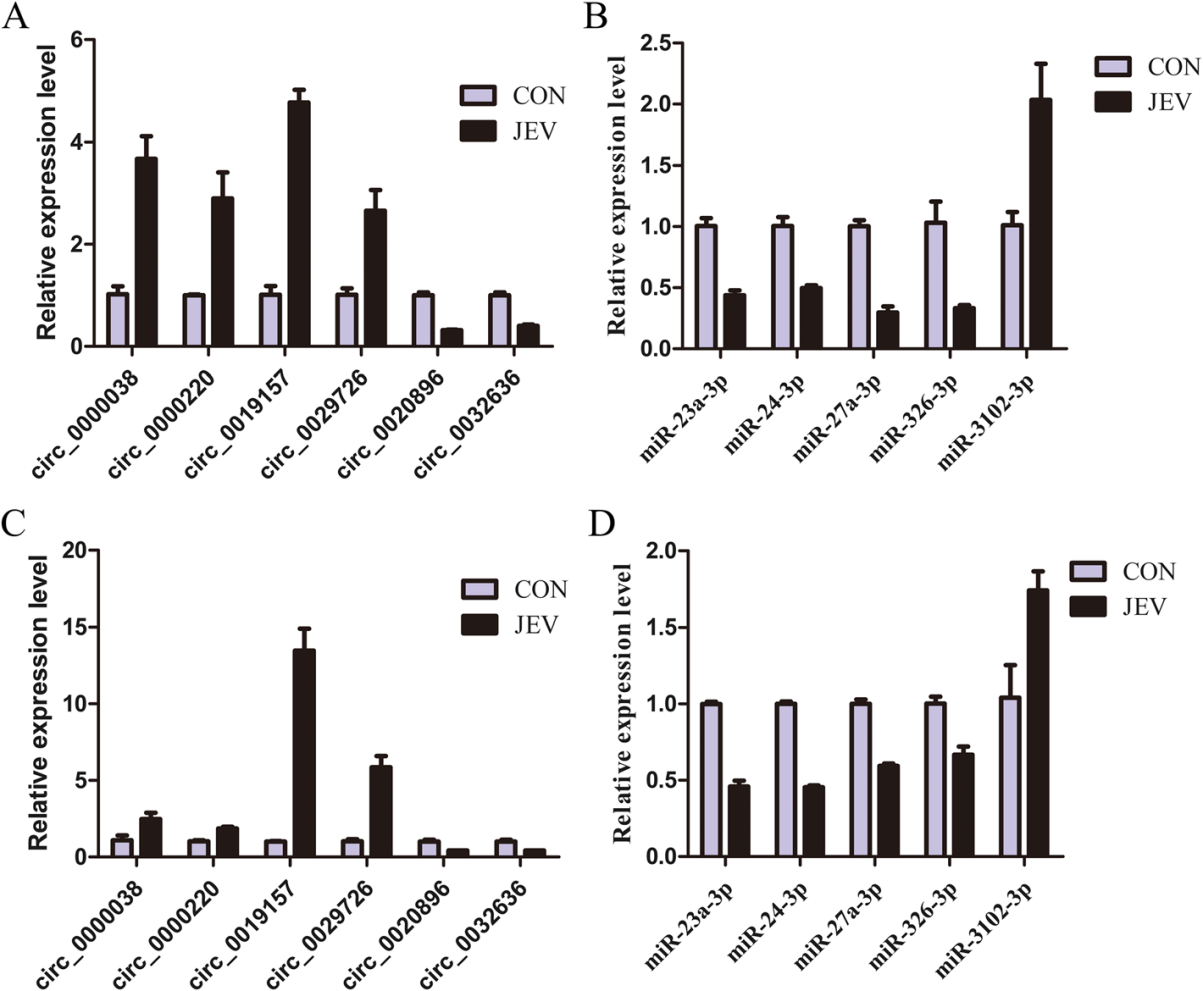
Validation of the sequencing data using the quantitative real-time PCR. The relative expression levels of circRNAs (**a**) and miRNAs (**b**) measured in JEV-infected or mock-infected mice brains. Cultured wild-type BV2 cells were infected with JEV at MOI of 5 for 24 h, and the relative expression level of circRNAs (**c**) and miRNAs (**d**) were measured. Data are expressed as mean ± SEM from three independent experiments

To validate the expression levels of JEV-induced circRNAs and miRNAs found in our sequencing data, we randomly selected six differentially expressed circRNAs and five differentially expressed miRNAs, and validated their expression in JEV-infected or mock-infected mice brain tissues by quantitative real-time PCR. The expression patterns were analogous to those as observed in our sequencing data (Fig. 5a, b). Furthermore, we examined the expression levels of these circRNAs and miRNAs in cultured mouse microglia (BV2 cells). As expected, these circRNAs and miRNAs showed similar expression patterns as were observed in mice brain tissues (Fig. 5c, d).

### CircRNA_0000220 positively regulates the production of JEV-mediated inflammatory cytokines and acts as a ceRNA of miR-326-3p

**Fig. 6:**
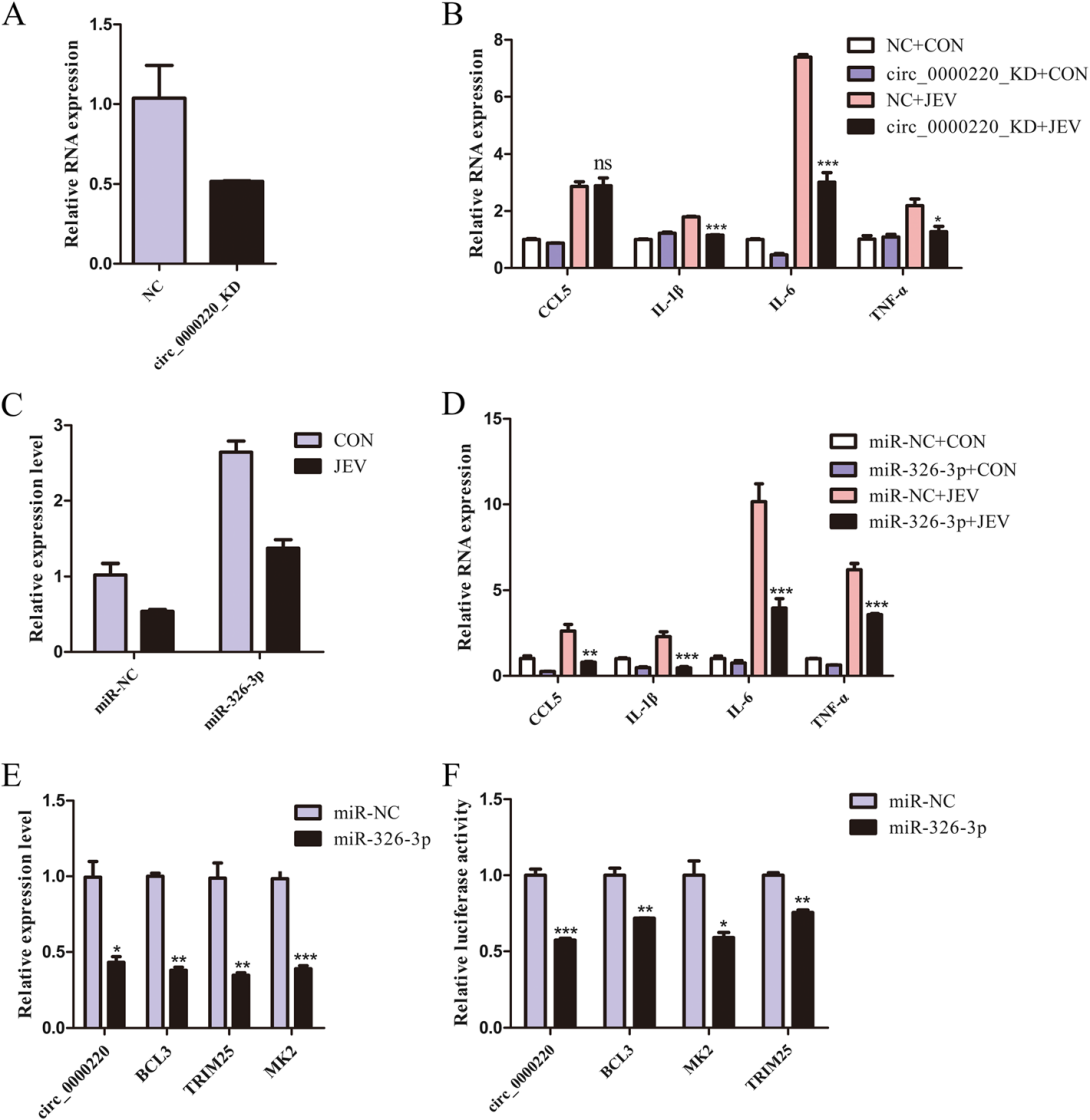
CircRNA_0000220 positively regulates the production of JEV-mediated inflammatory cytokines and acts as a ceRNA of miR-326-3p. **a** The relative expression level of circ_0000220 measured in CRISPR/Cas9 knockdown BV2 cells (circ_0000220_KD) or wild-type BV2 cells (NC). **b** Circ_0000220 knockdown BV2 cells or wild-type BV2 cells were infected with JEV at MOI of 5 MOI. At 24 h post-infection, samples were collected and mRNA expression levels of TNF-α, IL-6, IL-1β, and CCL5 were measured by quantitative real-time PCR. **c** and **d** The wild-type BV2 cells were transfected with miR-326-3p mimics or mimics control (miR-NC) for 24 h, followed by infection with JEV at 5 MOI. At 24 h post-infection, the relative mRNA expression levels of miR-326-3p (**c**) and TNF-α, IL-6, IL-1β, and CCL5 (**d**) were measured by quantitative real-time PCR. **e** The wild-type BV2 cells were transfected with miR-326-3p mimics or mimics control (miR-NC) for 24 h. The relative expression levels of circ_0000220 and mRNAs (BCL3, MK2, and TRIM25) were measured by quantitative real-time PCR. **f** HEK-293 T cells were transfected with miR-326-3p mimics or mimics control (miR-NC) together with luciferase reporter plasmid harboring the 3′ UTR of circ_0000220, BCL3, MK2, or TRIM25 for 24 h, and followed by measurement of the luciferase activity. Data are expressed as mean ± SEM from three independent experiments

To interrogate the role of cirRNAs in the JEV-induced inflammatory response, we selected one of the differentially expressed circRNAs, the circ_0000220, predicted to be involved in inflammation. Since microglia are permissive to JEV infection and trigger a robust inflammatory response upon JEV infection [5], we chose BV2 cells for further analyses. First, we successfully constructed the circ_0000220 knockdown BV2 cells by employing the CRISPR/Cas9 method (Fig. 6a), and subsequently, used these cells to evaluate the inflammatory response produced upon JEV infection. It was observed that the silencing of circ_0000220 followed by JEV infection resulted in a significantly reduced production of inflammatory cytokines as assessed by quantitative real-time PCR (Fig. 6b). Considering the circRNAs as miRNAs sponges [16], the up- and down-regulation of circ_0000220 and miR-326-3p, respectively, in our data, the functional enrichment of circ_0000220 and miR-326-3p in the inflammation, we speculated that circ_0000220 may positively regulate JEV-mediated inflammatory response via miR-326-3p. To this end, cultured wild-type BV2 cells were transfected with synthetic miR-326-3p mimics or mimics control and then infected with JEV. Overexpression of miR-326-3p caused a significantly diminished expression of inflammatory cytokines (Fig. 6c, d). Furthermore, ectopic expression of miR-326-3p led to a markedly down-regulated expression of circ_0000220 and miR-326-3p-target mRNAs (BCL3, MK2, and TRIM25), determined through quantitative real-time PCR and dual-luciferase assay (Fig. 6e, f). Taken together, these data suggest that circ_0000220 positively regulates the JEV-induced inflammatory response, and this observed effect may occur via sponging the miR-326-3p and by subsequently affecting the expression of miR-326-3p-target mRNAs.

## Discussion

The genome-wide transcriptomic approaches are becoming a powerful tool to dissect the intricate host-virus interactions. Several studies have highlighted the role of non-coding RNAs (long non-coding RNAs and miRNAs) in orchestrating the cellular processes altered upon infection with several viruses, including JEV [19, 25]; however, the signatures and functions of relatively a new class of non-coding RNAs, called circRNAs, during viral infections remain largely unknown. Thus, it is intriguing to examine the expression profile and interaction of circRNAs with other endogenous RNAs. Herein, the genome-wide RNA-sequencing of circRNAs and miRNAs from JEV-infected mice brain tissues was performed. The findings obtained from this study indicated for the first time the intricate regulation of circRNAs during JEV infection and suggests their potential role in JEV neuropathogenesis.

Recent studies demonstrated that circRNAs are preferentially spliced and expressed abundantly in brain tissues [26, 27]. The expression of circRNAs is also evolutionary conserved from mouse to human, highlighting their functions during brain development [28]. Given the role of circRNAs in brain physiology and the tendency of JEV to infect the brain, we surmised that circRNAs may participate in regulating the neuroinflammatory response triggered upon JEV infection. Our RNA-sequencing data analyses identified 180 circRNAs and 58 miRNAs differentially regulated in JEV-infected mice brain tissues. By employing biological and pathway enrichment analyses, these circRNAs were found to be associated with brain signaling, epigenetic modifications, cellular transcription, and inflammation signaling. The CRISPR-Cas9-mediated knocking down of a selected circRNA, the circ_0000220, in cultured mouse microglial cells validated its function in regulating the production of inflammatory cytokines during JEV infection, suggesting a potential therapeutic target to ameliorate the signs of JEV-induced encephalitis. Whether or not, the targeting of circ_0000220 offers a therapeutic effect against Japanese encephalitis in vivo, remains to be elucidated in future studies.

During the transcriptomic analysis of our data, we found that most of the identified circRNAs exhibit exonic origin, which is consistent with previous studies [7]. Upon comparison of our circRNAs sequences with the circBase database, we found that only ~ 50% of the detected circRNAs from each experimental replicate were pairable with the circBase sequences, indicating that half of the circRNAs identified in our study are non-annotated which could be novel or may reflect tissue/cell specificity. Thus, our study suggests the need for enrichment of the circBase database to advance the research related to circRNAs.

It has been widely reported that circRNAs act as miRNA sponges to regulate various biological processes [16]. The circRNA ciRS-7 by sponging miR-7 [22] regulates the onset of Alzheimer’s disease [29]. The ciRS-7/miR-7 axis controls the proliferation, apoptosis, and inflammation of the chondrocytes [30]. Moreover, the circRNA circHIPK3 though interacting with miR-124 regulates the neuroinflammation in diabetic rats [31]. Our established ceRNA interaction network uncovered several new interactions that may involve in JEV-induced inflammatory response. Among them, circ_0000220 and miR-326-3p were confirmed to regulate the production of inflammatory cytokines during JEV infection, possibly by controlling the expression of BCL3, MK2, and TRIM25 mRNAs. Further studies are required to understand the correlation between circRNAs and other endogenous RNAs in more detail.

## Conclusions

In summary, we first unveiled the signatures of circRNAs and related miRNAs in the JEV-infected mice brain using the high throughput RNA-sequencing method. By employing the bioinformatic algorithms, we predicted that some of these circRNAs may regulate the neuroinflammatory response during JEV infection. The experimental validation of the involvement of selected circRNA during the JEV-induced inflammatory response confirmed our predictions. This study may advance the understanding of the molecular aspects of the JEV neuropathogenesis. Since the roles of circRNAs during viral infections is still at its infancy, our study may serve as a resource for future studies.

## Methods

### Cell and virus

Mouse microglial cells, baby hamster kidney (BHK-21) cells, and human embryonic kidney (HEK293T) cells were cultured and maintained in Dulbecco modified Eagle medium (DMEM; Sigma) supplement with 10% fetal bovine serum (FBS; Gibco), 100 U/ml penicillin, and 100 mg/ml streptomycin sulfate at 37 °C in 5% CO2 atmosphere. The circRNA knockdown BV2 cells were engineered by the CRISPR-Cas9 system. Knockdown cells were grown under conditions similar to that of naïve cells but appended with 2 μg/ml puromycin (InvivoGen). The JEV P3 strain used in this study was propagated in our laboratory.

### JEV infection

Adult 6-weeks-old BALB/C mice were purchased from Hubei Provincial Center for Disease Control and Prevention, Wuhan. Mice were randomly divided into two groups (*n* = 3/group): JEV-infected group and control group. Mice belonging to the JEV-infected group were intracerebrally injected with 200 plaque-forming units (PFU) of JEV P3 strain in 20 μl DMEM, whereas mice assigned to control group were injected intracerebrally with an equal amount of DMEM. On day 5 after infection, mice infected with JEV exhibited the signs of acute encephalitis. Mice from both groups were anesthetized with 2% isoflurane, followed by cervical dislocation, and the brain tissues were collected for further experiments. All animal experiments were performed following the National Institute of Health Guide for the Care and Use of Laboratory Animals, and the experimental protocols were approved by the Research Ethics Committee of College of Veterinary Medicine, Huazhong Agricultural University, Hubei, Wuhan, China (No. S02914040M). All types of virological assays were performed under Biosafety Level 2 containment. We performed a double-blind procedure in our in vivo experiments by including blinding investigators, participants, and outcome assessors.

### RNA extraction and quantitative real-time PCR

Total RNA extraction was performed using TRIzol reagent (Invitrogen). To analyze mRNA expression, 1 μg of total RNA was reverse transcribed into cDNA using First Strand cDNA Synthesis Kit (TOYOBO) following the manufacturer’s instructions. For the expression analysis of circRNA, linear RNAs were removed by RNase R treatment, and the circRNA-specific outward-facing divergent primers were designed for subsequent experiments. Quantitative real-time PCR was performed using a ViiA™ 7 Real-time PCR System (Applied Biosystems) and SYBR Green Real-time PCR Master Mix (TOYOBO). Amplification was performed for 2 min at 50 °C and 10 min at 95 °C, followed by 40 cycles of 95 °C for 15 s, 60 °C for 15 s, and 72 °C for 30 s. The relative expression levels of mRNA and circRNA were normalized to that of β-actin within each sample using the 2^−ΔΔCt^ method.

For miRNA expression, a commercial Bulge-Loop™ miRNA quantitative reverse transcription (RT)-PCR detection method was used. Briefly, 1 μg total RNA was used as the template and reverse transcribed using a miRNA specific RT primer. The resulting cDNA was used for quantitative real-time PCR with a universal reverse primer and a specific forward primer. The relative expression levels of miRNA were normalized to that of U6 within each sample using the 2^−ΔΔCt^ method. The specific primers for the detection of mRNA, circRNA, and miRNA are listed in Additional file 7: Table S6.

### CircRNA sequencing and analysis

The total RNA isolated from mice brain tissues was treated with the Ribo-Zero™ rRNA Removal Kit (Illumina) and RNase R to deplete rRNA and linear RNA, respectively. The remaining RNA was fragmented and subsequently reverse transcribed using random hexamer oligos. The cDNA library containing 300 ~ 400 bp fragments was constructed by the TruSeq RNA Sample Prep Kit (Illumina) and sequenced on the Illumina HiSeq4000 sequencer (Illumina) in 150 bp paired-end reads. Adaptor sequences and low-quality ends per reads were trimmed using the Trimmomatic v0.39 with the following options: minimum quality score of 20 in both ends per read, a minimum mean quality score of 20 in a sliding window of 4 bp, and a minimum read length of 50 bp [32]. Quality control of raw reads was performed by using the FastQC v0.11 (http://www.bioinformatics.babraham.ac.uk/projects/fastqc/). The expression of circRNAs was detected and quantified using the CIRI v2.0.6 algorithm for back-splice identification [33]. Briefly, the sequencing reads spanning back-spliced junctions were extracted from the reads unmapped to the mouse reference genome mm10. To eliminate false positives, the circRNA candidates were initially filtered with at least two back-spliced reads as suggested previously [34]. We further filtered out the circRNA candidates from repetitive genomic regions through the UCSC table browser (http://genome.ucsc.edu/cgi-bin/hgTables), and the spliced sequences of circRNAs were extracted through the circPrimer software [35]. Default parameters were used unless otherwise stated above. The circRNA raw data has been deposited in NCBI Sequence Read Archive under accession number SRP254062.

### Small RNA sequencing and analysis

The small RNA library for sequencing was prepared by using the TruSeq Small Prep Kit Preparation (Illumina). Samples were sequenced on the Illumina HiSeq2000 sequencer (Illumina) in 50 bp single-end reads. Reads shorter than 17 bp and longer than 35 bp were removed through the Trimmomatic v0.39 software [32]. The remaining reads were aligned to the mouse miRNA precursors, allowing no mismatches, downloaded from the miRbase v22 [36]. In total, the dataset contains 1978 mature miRNA sequences of the mouse. Reads uniquely aligned to miRNAs were retained and counted. The miRNAs in sRNA sequencing data were identified using the miRDeep2 v2.0 [37]. The small RNA sequencing raw data has been deposited in NCBI Sequence Read Archive under accession number SRP253986.

### Differential expression analysis

Based on the above analyses, read counts were obtained for each RNA entity (circRNAs and miRNAs) across samples. The Bioconductor package DESeq2 v1.24.0 was employed to estimate the differential expression of circRNAs and miRNAs [38]. To remove very low-expressed and/or un-expressed candidates, genes with counts less than 10 and transcript-per-million (TPM) values less than 1 in more than 50% of all samples were filtered out from the two groups. The R package limma v3.40.6 was used to estimate the differential expression of genes/mRNAs [39]. Differentially expressed RNA entities were selected based on the following criteria: |log2 fold change| > = 1 and FDR < 0.05. The heatmap and volcano plots were generated using the R package pheatmap v1.0.12 and ggplot2 v3.2.1 for visualizing the different genes [40, 41].

### Gene-set enrichment analysis

Gene Ontology (GO) term and Kyoto Encyclopedia of Genes and Genomes (KEGG) pathway enrichment analyses of differentially expressed circRNAs were carried out by using the R package clusterProfiler v3.12.0 [42].

### Construction of the circRNA–miRNA–mRNA network

To investigate miRNAs interactions with mRNAs and circRNAs, the miRNA binding sites on these RNA entities were predicted by the miRanda v3.3a, an algorithm recognizing target sites based on sequence complementarity between miRNAs and the free energy of RNA duplex [43]. By combining the differentially expressed circRNAs and miRNAs identified in this study, with differentially expressed mRNAs from our previous study [19], we constructed a ceRNA interaction network and displayed results using the Cytoscape v3.7.1 [44].

### Dual-luciferase assay

The circRNA and 3′ UTR of mRNAs were cloned into the psiCheck-2 plasmid to carry out the dual-luciferase assay. Cultured HEK-293 T cells were transfected with 200 ng plasmids along with miRNA mimics, inhibitors, or controls. After 36 h, the transfected cells were collected to measure the firefly and Renilla luciferase activities using the dual-luciferase reporter system (Promega).

### Statistical analysis

All results are expressed as mean ± SEM. Statistical analyses were performed by using the GraphPad Prism 5 software. Statistical significance was determined with a two-sided unpaired t-test for two groups or multiple comparisons one-way of variance (ANOVA) for more than two groups (Bonferroni’s multiple comparison test, *P* < 0.05).

## Supplementary information

**Additional file 1: Table S1.** The detail information of identified circRNAs.

**Additional file 2: Table S2.** Differentially expressed circRNAs between JEV-infected and mock-infected groups.

**Additional file 3: Figure S1.** Summary of the Gene Ontology (A) and KEGG pathway (B) analysis for the parental genes of the differentially expressed circRNA parent genes.

**Additional file 4: Table S3.** The GO and KEGG analysis of differently expressed circRNA parent genes.

**Additional file 5: Table S4.** Differentially expressed miRNAs between JEV-infected and mock-infected groups.

**Additional file 6: Table S5.** The circRNA-miRNA-mRNA ceRNA network.

**Additional file 7: Table S6.** The specific primers for mRNAs, circRNAs, and miRNAs.

## Data Availability

All the RNA-seq data are available on the available in the NCBI Sequence Read Archive (SRA) repository with the accessions SRP254062 for circRNAs, SRP253986 for miRNAs.

## Abbreviations

JEV: Japanese encephalitis virus
ncRNA: Noncoding RNA
CircRNAs: Circular RNAs
miRNAs: microRNAs
ceRNA: Competing endogenous RNA
PFU: Plaque forming units
TPM: Transcript per million
GO: Gene Ontology
KEGG: Kyoto Encyclopedia of Genes and Genomes
DMEM: Dulbecco’s Modified Eagle Medium
FBS: Fetal bovine serum
FC: Fold change
FDR: False discovery rate
