# Supplementary figures and images for: Genome-wide profiling of host-encoded circular RNAs highlights their potential role during the Japanese encephalitis virus-induced neuroinflammatory response
Source: BMC Genomics. 2020 Jun 18;21:409. doi: 10.1186/s12864-020-06822-5 (PMC7301528; doi:10.1186/s12864-020-06822-5)

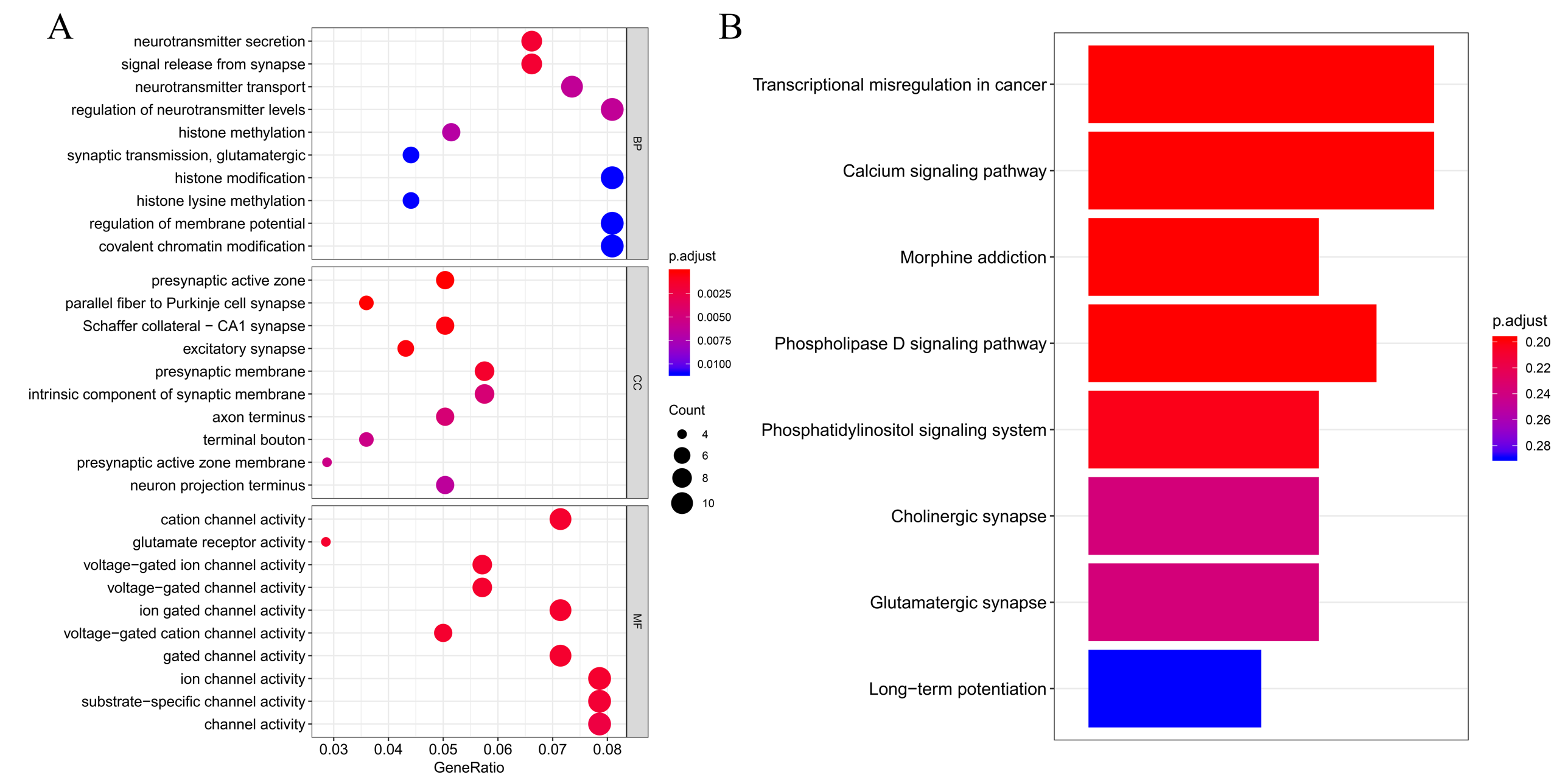

Supplement: Supplementary file 3 — Additional file 3: Figure S1. Summary of the Gene Ontology (A) and KEGG pathway (B) analysis for the parental genes of the differentially expressed circRNA parent genes. [file 12864_2020_6822_MOESM3_ESM.tif]
